# Are we making progress in the diagnosis and management of sarcopenia? Results from a UK-wide survey

**DOI:** 10.1007/s41999-025-01321-w

**Published:** 2025-10-07

**Authors:** Christopher Hurst, Claire McDonald, Rachel Cooper, Avan A. Sayer, Miles D. Witham

**Affiliations:** 1 AGE Research Group, Faculty of Medical Sciences, Translational and Clinical Research Institute, Newcastle University, Newcastle upon Tyne, UK; 2 NIHR Newcastle Biomedical Research Centre, Newcastle upon Tyne Hospitals NHS Foundation Trust, Cumbria, Northumberland, Tyne and Wear NHS Foundation Trust and Faculty of Medical Sciences Newcastle University, Newcastle upon Tyne, UK

**Keywords:** Sarcopenia, Survey, Older people, Diagnosis

## Abstract

**Purpose:**

To describe current practice in diagnosis and management of sarcopenia in older people in the United Kingdom (UK).

**Methods:**

Healthcare professionals from UK organisations were invited to complete an online survey about sarcopenia diagnosis and management.

**Results:**

One hundred and one responses were analysed. Respondents represented a range of professional groups across 62 organisations from secondary care, primary care, community providers and private practitioners. Sarcopenia was identified in only 32/62 (52%) organisations. Within these organisations, 17/32 (53%) did not use a recognised algorithm for diagnosis, although 31/32 (97%) of organisations assessed muscle strength. Twenty-nine (91%) organisations reported offering interventions to people diagnosed with sarcopenia, but resistance training was offered by only 19 (59%).

**Conclusion:**

This survey has shown that just over 50% of respondent organisations in the UK are identifying sarcopenia in older people as part of clinical practice, highlighting the need for ongoing work to achieve further improvement.

**Supplementary Information:**

The online version contains supplementary material available at 10.1007/s41999-025-01321-w.

## Key summary points

**Aim:**

To describe current practice in the diagnosis and management of sarcopenia in older people in the United Kingdom (UK).

**Findings:**

This new survey of practice in the UK showed that half of the responding organisations reported diagnosing sarcopenia. Among organisations that diagnosed sarcopenia, around half used established consensus guidelines and nearly two-thirds offered resistance exercise training.

**Message:**

There has been a modest increase in evidence-based diagnosis and management of sarcopenia in the UK compared to previous survey results highlighting the need for ongoing efforts to deliver improvements in the diagnosis and evidence-based treatment of sarcopenia in older adults.

**Supplementary Information:**

The online version contains supplementary material available at 10.1007/s41999-025-01321-w.

## Introduction

Sarcopenia is “a progressive and generalised skeletal muscle disorder involving the accelerated loss of skeletal muscle mass and function” [1]. It is estimated to affect 1 in 10 older people in the general population with a much greater prevalence amongst those living in care [2]. Sarcopenia leads to a loss of mobility and physical function and an increased risk of adverse health outcomes, including falls, fractures and premature mortality [1, 2]. Despite this, there remains a lack of knowledge and awareness of this condition in clinical practice [3, 4]. This presents a challenge as without wider recognition and case finding, interventions such as resistance exercise training [5], cannot be initiated and/or targeted at those with greatest need.

Several operational definitions exist to support the diagnosis of sarcopenia [6–8]. However, a previous survey in 2018 found that many services (47%) specialising in the care of older people in the United Kingdom (UK) did not make the diagnosis of sarcopenia and most did not routinely offer appropriate treatment [9]. Since then, sarcopenia has received increasing attention in research and clinical practice, with initiatives ongoing to support the improved management of sarcopenia [10]. The aim of this work was to survey healthcare professionals to provide an up-to-date understanding of practice in the diagnosis and management of sarcopenia in the UK.

## Methods

We conducted an online survey to evaluate current practice in the diagnosis and management of sarcopenia in the UK. The survey included questions focused on the identification of sarcopenia, diagnostic criteria used, and the interventions offered to individuals diagnosed with sarcopenia (see Supplementary Table 1). To facilitate comparison over time, the core questions were replicated from our previous national survey conducted in 2018 [9]. Additional items were included in the 2025 survey to explore the coding and communication of sarcopenia diagnosis. Data were collected using Microsoft Forms.

We sought to target members of multiprofessional teams with responsibility for providing care for older people with and at risk of sarcopenia. As in 2018, the survey was disseminated via the mailing lists of the British Geriatrics Society Sarcopenia and Frailty Research Special Interest Group (SiG) and promoted on social media platforms X (formerly Twitter), Bluesky and LinkedIn. To increase engagement and representation across all relevant professional groups the 2025 survey was additionally circulated through AGILE (Chartered Society of Physiotherapists working with Older People), the British Dietetic Association Older People Specialist Group, and the ART (Ageing Research Translation) of Healthy Ageing network. The survey was promoted at a British Geriatrics Society national conference in Autumn 2024. The questionnaire was launched on 18 November 2024 and closed on 21 January 2025.

As in the previous UK-based survey [9], where there were multiple respondents from the same organisation, responses were combined to enable analysis at the level of the responding organisation. Where respondents from within the same organisation had provided conflicting responses, if at least one respondent indicated a positive response (e.g., that sarcopenia was identified), the organisation was coded as having this response. Following UK Health Research Authority (www.hradecisiontools.org.uk) guidance, the project was not classified as research and so did not require research ethics approval. Raw data from Microsoft Forms were downloaded and analysed using Microsoft Excel, with data presented as descriptive statistics.

## Results

Over a period of 10 weeks, 115 survey responses were received. Of these, 101 were eligible for inclusion in analyses. Fourteen responses were excluded (10 outside the UK, three insufficient information provided to determine location or clinical affiliation and one duplicate). Respondents included a mix of geriatricians (30%), physiotherapists (38%), advanced clinical practitioners (12%) and others (see Supplementary Table 2 for full list of professional groups). Only 10% of the respondents had completed the 2018 survey. Respondents represented 62 organisations from across England, Scotland, Wales and Northern Ireland. Most of the organisations represented (49/62; 79%) were NHS trusts or Scottish or Welsh Health Boards, with primary care, community providers and private practitioners also represented.

Respondents from thirty-two different organisations (52%) reported that sarcopenia was identified in older people as part of clinical practice in their organisation; 17 organisations identified sarcopenia in both outpatients and inpatients, 12 in outpatients/community only and three in inpatients only. Within the 32 organisations identifying sarcopenia, at least one measure of muscle strength was used to diagnose sarcopenia by 31 (97%), a measure of muscle mass was used by 13 (41%), physical performance measures were used by 25 (78%) and other diagnostic criteria were used by two (6%). In fifteen organisations (47%), a diagnostic algorithm for sarcopenia was used; the European Working Group on Sarcopenia in Older People 2 (EWGSOP2) diagnostic criteria in 11 (34%), and an unspecified algorithm in four others. A range of tools were used to measure relevant sarcopenia criteria (Table 1), the most common being grip strength, which was assessed by 25/32 (78%) organisations that identified sarcopenia. No organisation reported using ultrasound to assess muscle mass.

**Table 1:** Tools used to measure different diagnostic criteria for sarcopenia

Diagnostic criteria	Tool used	Number (%) of organisations*
Muscle strength	Grip strength	25 (78)
	Chair stand test	24 (75)
	Grip strength & chair stand test	20 (63)
Muscle mass	Bioimpedance	1 (3)
	DXA	9 (28)
	Computed Tomography	3 (9)
	Magnetic Resonance Imaging	2 (6)
	Anthropometry	9 (28)
Physical performance	Short physical performance battery	4 (13)
	Walking speed	23 (72)
Questionnaire	SARC-F	11 (34)
Other		4 (13)

*DXA* Dual energy X-ray Absorptiometry. *SARC-F* Strength, Assistance, Rise, Climb – Falls

^*^Denominator for calculation of percentages = all organisations who reported identifying sarcopenia (*n* = 32)

Twenty-nine organisations (91%) reported that their organisation offered interventions to people diagnosed with sarcopenia and resistance exercise training (59%) and functional exercise training (81%) were the most common (Table 2). Of the 32 organisations diagnosing sarcopenia, there were 8 (25%) that included sarcopenia on inpatient discharge summaries and 18 (56%) that included sarcopenia as a diagnosis on outpatient correspondence.

**Table 2:** Interventions offered to people diagnosed with sarcopenia

Interventions offered	Number (%) of organisations*
Exercise	
Resistance exercise training	19 (59)
Functional exercise training	26 (81)
Other exercise training	6 (19)
Nutrition and Supplementation	
Vitamin D	17 (53)
Protein supplementation	10 (31)
Other nutritional interventions	12 (38)
Drugs	2 (6)
Other	4 (13)

^*^Denominator for calculation of percentages = all organisations who reported identifying sarcopenia (*n* = 32)

## Discussion

Our survey shows that half of responding organisations in the UK diagnose sarcopenia. Of the organisations that do, nearly all measure muscle strength and almost half use a recognised diagnostic algorithm. Around two-thirds of organisations diagnosing sarcopenia offer resistance exercise, the current evidence-based standard of care. The data also demonstrate evidence that sarcopenia is being identified across primary, secondary and community care settings with diagnosis and treatment delivered by a broad range of health care professionals, including medical, nursing and allied health care practitioners, reflecting the multidisciplinary approach to geriatric medicine prevalent in the UK.

Although the proportion of organisations identifying sarcopenia has remained relatively stable compared with the previous UK survey (52% [32/62] vs 53% [26/49]) [9] our data show an increase in the use of a recognised algorithm (47% vs 12%) suggesting improved standardisation in how sarcopenia is diagnosed [9]. We do not know the diagnostic approaches employed by organisations not using recognised algorithms, and further investigation is needed to fully understand clinical practice across the UK. However, our findings are consistent with a previous European survey, which found that several tools are currently used to assess muscle mass, muscle strength, or physical performance [13] and that lack of knowledge and availability of diagnostic equipment remain barriers to the diagnosis of sarcopenia in clinical practice [14]. Our findings suggest that UK practitioners already demonstrate greater familiarity with recognised diagnostic tools for sarcopenia compared with clinicians in the USA, where recent data indicate limited awareness of sarcopenia diagnostic criteria [4]. The lack of use of ultrasound to assess muscle mass likely reflects reservations around its practical use and reliability outside a research setting. Together these data suggest there is an international need for a broader package of support to assist those working in clinical practice to diagnose sarcopenia. Previous efforts to support and enhance the delivery of resistance exercise training in clinical practice through clear guidance [5] and quality improvement [10], could be replicated with an increased focus on the diagnosis of sarcopenia in clinical practice.

Encouragingly, efforts to enhance the delivery of resistance exercise in the UK appear to be yielding positive results. The survey found that the proportion of organisations offering resistance exercise training to their patients was higher than reported in previous UK surveys [9, 11]. Resistance exercise training is the only intervention with a robust evidence base to support its use as a treatment for sarcopenia and older people are willing to engage if they are appropriately supported [12]. Interestingly several organisations reported providing interventions lacking robust evidence, and a small number are administering pharmacological treatments despite the absence of any drugs currently approved for sarcopenia.

For resistance exercise to be effective it must be sustained. The survey demonstrates that organisations that diagnose and treat sarcopenia do not consistently record the diagnosis in clinical summaries or letters. This omission can lead to fragmented care, hinder continuity across services, and compromise monitoring and follow-up. From a public health perspective, it limits effective service planning and impedes the identification of population-level trends. From a research perspective, it presents significant barriers to identifying eligible patients for clinical trials or observational studies, thereby hindering recruitment efforts and the generation of high-quality evidence to support emerging therapies.

### Strengths and limitations

Until the assessment and diagnosis of sarcopenia is routinely recorded in medical records this survey presents the most reliable way to assess current practice in the UK. However, a number of potential limitations should be noted. Participation bias may limit the representativeness of our findings, as clinical practitioners responding to the survey may have a greater interest in sarcopenia than those who did not. Secondly, our survey design was based on a previous UK survey conducted in 2018 to enable us to draw comparisons across two time points. However, only 10% of respondents to the present survey had completed the previous survey, possibly reflecting differences in sampling approaches. This makes it difficult to draw firm conclusions about change over time. It is, however, encouraging to note that we obtained a greater number of responses in this survey compared with the previous one (101 vs. 61 respondents), with a higher number of organisations represented (62 vs. 49). The broad range of professional groups participating is also a strength, reflecting the multidisciplinary nature of care for older people in the UK. This is particularly important given that previous international surveys have identified differences in practice between professional groups [4, 15]. However, the relatively modest sample size limited our statistical power to explore variations in responses by profession or region.

In conclusion, our findings highlight growing standardisation in sarcopenia diagnosis and increased delivery of evidence-based treatment across UK healthcare settings. However, only half of responding organisations are currently diagnosing sarcopenia, and inconsistent documentation and varied diagnostic approaches remain key barriers. Future efforts should focus on supporting clinical teams with practical tools, training, and system-level changes to embed routine diagnosis and long-term management of sarcopenia. Qualitative work exploring how teams work together to make decisions about sarcopenia measurement, diagnosis and treatment would also assist these efforts.

## Supplementary Information

Below is the link to the electronic supplementary material.

Supplementary file1 (DOCX 21 KB)

## Data Availability

The data from this study are available upon reasonable request from the corresponding author.
