# Supplementary material for: Are we making progress in the diagnosis and management of sarcopenia? Results from a UK-wide survey
Source: Eur Geriatr Med. 2025 Oct 7;17(2):1059–63. doi: 10.1007/s41999-025-01321-w (PMC13109140; doi:10.1007/s41999-025-01321-w)
Supplement: Supplementary file 1 — Supplementary file1 (DOCX 21 KB) [file 41999_2025_1321_MOESM1_ESM.docx]

**Are we making progress in the diagnosis and management of sarcopenia? Results from a UK-wide survey**

European Geriatric Medicine

Christopher Hurst^1,2^, Claire McDonald^1,2^, Rachel Cooper^1,2^, Avan A Sayer^1,2^, Miles D Witham^1,2^

1. AGE Research Group, Translational and Clinical Research Institute, Faculty of Medical Sciences, Newcastle University, Newcastle, UK
2. NIHR Newcastle Biomedical Research Centre, Newcastle upon Tyne Hospitals NHS Foundation Trust, Cumbria, Northumberland, Tyne and Wear NHS Foundation Trust and Faculty of Medical Sciences Newcastle University, Newcastle upon Tyne, UK

**Corresponding author:**

Professor Miles Witham

Miles.Witham@newcastle.ac.uk

**Supplementary material**

1. Supplementary Table 1: Survey questions
2. Supplementary Table 2: Professional role of questionnaire respondents

| Supplementary Table 1. Survey questions |
| --- |
| 1. First name 2. Last name 3. Organisation 4. Your role in the organisation 5. Did you complete our survey in 2018? 6. In your organisation, do you or your colleagues identify sarcopenia in older people? 7. Which tools do you or your colleagues use to diagnose sarcopenia? (please select all that apply) 8. What diagnostic criteria do you or your colleagues use to diagnose sarcopenia? (please select all that apply) 9. Please tell us which algorithm you or your colleagues use to diagnose sarcopenia: 10. Do you or your colleagues offer any interventions to patients diagnosed with sarcopenia (e.g. exercise, nutrition, drugs)? 11. What interventions do you or your colleagues offer to patients diagnosed with sarcopenia? (please select all that apply) 12. Do you or your colleagues include sarcopenia as a diagnosis on inpatient discharge summaries? 13. Do you or your colleagues include sarcopenia as a diagnosis on outpatient correspondence? 14. Any comments or alternative pathways of care you can tell us about? Please give any additional details on diagnosis or management not covered by the questions |

| Supplementary Table 2. Professional role of questionnaire respondents | |
| --- | --- |
| Professional role | Number of respondents (n = 101) |
| Consultant geriatrician | 15 |
| Non-consultant career grade doctor | 4 |
| Trainee Geriatrician | 11 |
| General Practitioner (GP) | 4 |
| Nurse | 9 |
| Physiotherapist | 38 |
| Occupational therapist | 1 |
| Dietician | 4 |
| Other  Advanced Clinical Practitioner  Consultant Physician  Respiratory Registrar  Trust Grade Doctor | 12  1  1  1 |
